# Changes in the association between educational achievement, attainment and subsequent mental health: A survival analysis of 21 Swedish graduation cohorts

**DOI:** 10.1186/s12889-024-20554-1

**Published:** 2024-10-31

**Authors:** Björn Högberg, Mattias Strandh, Solveig Petersen

**Affiliations:** 1 Department of Social Work, Umeå University, Umeå, SE-901 87 Sweden; 2 Centre for Demographic and Ageing Research, Umeå University, Umeå, Sweden; 3 Department of Epidemiology and Global Health, Umeå University, Umeå, Sweden

**Keywords:** Performance, Education, School, Internalizing conditions, Internalizing disorders, Temporal trends

## Abstract

**Background:**

Low academic achievement and low educational attainment in adolescence is associated with higher risks of internalizing disorders later in life. However, less is known regarding if these associations vary over time across cohorts. The aim of this study was to investigate temporal changes in the association between academic achievement or educational attainment and subsequent inpatient treatment for internalizing disorders among Swedish youths.

**Methods:**

Register data on all students graduating from compulsory school in Sweden between 1990 and 2010 (*N* = 2 252 703) were used. Students were followed for a maximum of 8 years using discrete time proportional hazard models. Internalized disorders were measured by specialized inpatient psychiatric care for depression or anxiety disorders. Academic achievement was measured by grades at the end of compulsory school, and educational attainment by completion of upper secondary school.

**Results:**

The positive association between inpatient treatment for internalizing disorders and both low compulsory school achievement and non-completion of upper secondary school became stronger in more recent cohorts. The results were completely driven by girls and native-born youth.

**Conclusions:**

Low compulsory school achievements and failure to complete upper secondary school has become more important risk factors for inpatient treatment for internalizing disorders, particularly in native-born youth and girls. More research is needed to establish whether youth with internalizing disorders increasingly fail in school or whether low achievement has become more harmful for mental health.

**Supplementary Information:**

The online version contains supplementary material available at 10.1186/s12889-024-20554-1.

## Background

There is an educational gradient in mental health, such that students with lower grades and test results have higher risks of internalizing disorders [1–6]. This gradient is not only present in the short-term, but low academic achievement during the school-age years predicts internalizing disorders in youth and beyond [7–10]. Higher academic achievements may improve mental health directly by strengthening psychosocial resources such as self-esteem [11, 12]. Long-term benefits can also accrue due to higher attained education, which in turn can open doors to a range of socio-economic advantages such as higher incomes and social capital and lower unemployment risks [13–17].

Research has investigated the associations between achievement or attainment in adolescence and internalizing disorders later in life *within individuals in a given cohort* [8, 9, 18–20] but less is known about how these associations vary *over time across cohorts*. Both the structure of education systems and the function of education in society at large has undergone substantial changes in recent decades in ways that may influence how internalizing disorders and achievement or attainment interrelate.

The present study makes two primary contributions to the literature on academic achievement or educational attainment and mental health. First, only two studies to date, both using Swedish data, have investigated variation over time in the association between academic achievement and measures related to mental health later in life, with both finding stronger associations in more recent cohorts [7, 10]. Compared to these, we include a more recent and large sample, including all students (*N* = 2 252 703) graduating from compulsory school in Sweden between 1990 and 2010. The larger timespan allows for a more fine-grained picture of temporal variation, including non-linear changes, while the larger sample allows for more precise estimates of subgroup differences. Second, the literature on the educational gradient in mental health overall has primarily focused on the full adult population [13]. This study focuses on youth, who are often more dependent on their educational credentials for their labour market prospects due to limited work experience and less useful networks [21]. Young workers are also often among the most vulnerable in times of economic downturns and labour market restructuring [22], which in turn can have adverse mental health consequences [23].

The aim of the present study was to investigate temporal changes in the association between academic achievement or educational attainment and subsequent inpatient treatment for internalizing disorders among Swedish youths. The following specific research questions (RQs) were addressed:

### RQ 1

Has the association between academic achievement at the end of compulsory school and subsequent inpatient treatment for internalizing disorders within the following eight years changed across cohorts graduating between 1990 and 2010, and does this differ by immigrant background and sex?

### RQ 2

Has the association between upper secondary school completion and subsequent inpatient treatment for internalizing disorders within the following five years changed across cohorts graduating between 1990 and 2010, and does this differ by immigrant background and sex?

## Data and methods

Linked data from four Swedish administrative registers were used: the National Pupil Register, the Longitudinal Integrated Database for Health Insurance and Labour Market Studies (LISA), the National Patient Register, and the Total Population Register. Together, the registers contain information on academic achievement, subsequent educational attainment, treatment for psychiatric disorders, and basic demographic data. All students that graduated from year 9 of compulsory school (approximately 16 years old) between 1990 and 2010 were included and followed for a maximum of 8 years. The sample size with data on academic achievement and psychiatric treatment was 2 252 703. Data were made available by the Umeå SIMSAM Lab [24].

### Exposure variables – academic achievement and educational attainment

Academic achievement (RQ1) was measured using information on student’s school grades in year 9, the last year of compulsory school in Sweden. Students take 16 compulsory subjects in school – Swedish, mathematics, physical education, English, handicrafts, music, visual arts, technology, physics, chemistry, biology, history, social studies, religion, geography and home economics – as well as elective courses. The 16 or 17 subjects with the highest grades are added into an overall grade sum. This grade sum corresponds to a grade point average (GPA) and will henceforth be referred to as such. Grades are assigned locally by the student’s teachers, based on a detailed national set of grading criteria that is common for all schools. To further harmonize grading across schools, standardized national tests that anchor grades to national criteria are administered to all students, although some between-school variation in grading remains [25].

Year 9 GPA carries high stakes since it is used to allocate students to upper secondary school. If there are more applicants than slots in a school or program, students are accepted or rejected based on their year 9 GPA. If students do not attain a sufficient number of passing grades, they are not eligible to apply for national programs. Ineligible students are referred to remedial introductory programs that are often dead-ends and do not lead to an upper secondary diploma [26]. GPA was transformed into percentile ranks within graduation years to account for changes in the grading system and enhance comparability over time. The average association between GPA and inpatient treatment for internalizing disorders was curvilinear, with the 20% of students with the lowest GPA having especially high risk but with smaller differences within the rest of the GPA distribution (see Additional File A, Figure A1). To better understand the development for the most vulnerable youth, GPA was dichotomized with the bottom 20% coded 1 (= low GPA) and the top 80% coded 0 (= medium/high GPA), which is in line with related research [27, 28].

Subsequent educational attainment (RQ2) was operationalized as upper secondary school (UPS) non-completion. Upper secondary school follows directly from compulsory school and typically lasts for three years. Completion of this educational stage corresponds to attaining International Standard Classification of Education (ISCED) level 3, meaning education that either prepares for tertiary education or provides work-related vocational skills. UPS non-completion was measured by a time-varying indicator variable taking the value 1 if the individual had not completed an upper secondary school program in a given year, and 0 otherwise.

### Dependent variable – inpatient treatment for internalizing disorders

Internalizing disorders were measured using data on the primary diagnosis for treatment in psychiatric inpatient care. International Classification of Diseases 10 (ICD10) codes F30-39 for mood disorders and F40-44 for neurotic and stress-related disorders were used as indicators for internalizing disorders. ICD-9 was used until 1997, in which case codes 296, 298, 300, 311 were used for mood disorders and codes 300, 308, 309 for anxiety disorders. The variable was measured as time-varying and coded 1 for individuals that received treatment in psychiatric inpatient care at least once in a given year during follow-up, and 0 otherwise.

### Covariates

Inpatient treatment for internalizing disorders is a rare event, and graduation years were categorized into larger bins to improve precision. Three categories were used to balance the need for enough youths in each category and possibilities to investigate non-linear changes. Cohorts graduating between 1990 and 1997 were grouped into one reference category since formal eligibility requirements for upper secondary school were first introduced in 1998. The introduction of formal eligibility requirements disproportionately affected low-achieving students, many of whom were excluded from upper secondary education and subsequently from the labour market [26, 29, 30]. Later graduation cohorts were categorized as 1998–2004 and 2005–2010. This categorization does not mark any distinct change in the education system but ensured that there was comparable statistical power to detect significant effects in both groups.

Sex (0 = female; 1 = male) and native or immigrant background (0 = born in Sweden; 1 = born outside Sweden) were used as stratifying variables in models addressing RQ1 and RQ2. This is because previous research shows that the relationship between academic achievement or educational attainment and internalizing disorders may differ across these dimensions [31].

### Analytical strategy

Survival analysis techniques for discrete data and recurrent events were used. Participants were followed from the year of graduation until death, emigration, or the end of follow-up. The end of follow-up was defined as eight years after graduation from year 9 and was the same for all graduation cohorts. The average age of labour market entry for the studied cohorts was 22–24 years [32], or 6–8 years after the typical compulsory school graduation age. To the extent that low achievements constrain future employment prospects, most youths will have encountered these constraints by this stage.

Discrete time proportional hazard models were estimated to account for the yearly measurements of treatment for internalizing disorders. Participants may have experienced multiple treatment episodes during follow-up. Robust standard errors were estimated to account for the dependence of observations within individuals induced by the analyses of recurrent events [33]. Two groups of models were estimated. The first group of models addressed RQ1 and included graduation cohort, GPA, and interaction terms between graduation cohort and GPA. The second group addressed RQ2 and included graduation cohort, UPS non-completion, and interaction terms between graduation cohort and UPS non-completion. Since upper secondary school typically lasts for three years, the second group of models were restricted to three or more years after compulsory school graduation. Both groups of models were stratified by immigrant background and sex.

The models were first used to estimate hazard ratios depending on graduation year, GPA and their interaction. Hazard ratios are a measure on the relative scale and the interactions show the joint “effect” of graduation cohort and GPA or UPS non-completion, while taking differences in baseline risks into account. The models were then used to estimate predicted risks of inpatient treatment for internalizing disorders. Predicted risks can better convey between-group differences in absolute terms, which may have a greater public health relevance [34].

The aim of the study was descriptive and no confounders were adjusted for in the models [35].

### Supplementary and sensitivity analyses

Supplementary analyses were conducted to examine how sensitive the results were to specific modelling choices. A key assumption underlying discrete time proportional hazard models is the proportional hazards assumption, stating that the hazard of the event is constant over follow-up time for different levels of covariates. Figures B1-B4 in Additional file B indicate that the proportional hazards assumption was not fully supported in analyses involving UPS non-completion. This can bias confidence intervals and make the average hazard ratio a poor representation of the variation in the association over follow-up [36, 37]. Additional file B shows results from models using bootstrap methods to calculate confidence intervals, and models when coefficients are allowed to vary freely over follow-up time.

Comparisons of results from discrete time models fitted to different samples, in this case models for subgroups defined by immigrant background and sex, can reflect differences in unobserved heterogeneity across samples in addition to differential effects of the included variables [38]. The models were re-estimated using linear probability models, the estimates of which are not affected by differences in unobserved heterogeneity across samples.

The composition of the low GPA group may have changed due to immigration, with relatively more immigrant students among those with low grades. In Additional file D, low GPA was calculated separately for native-born and immigrant students.

Since UPS non-completion was a time-varying variable, the estimates may reflect reverse causation, that is, internalizing disorders causing changes in UPS completion. Reverse causation is not a source of bias given the descriptive aim of the study, but it may influence the interpretation of the results. In Additional File E, UPS completion was only measured three years after graduation from compulsory school, which is the typical age that Swedish youths complete upper secondary school.

Inpatient treatment for internalizing disorders is positively associated with mortality [39], making death a competing event that can prevent the main event of interest from occurring. Additional file F presents results from competing risk models [40], with all-cause mortality specified as a competing event.

To explore how sensitive the results were to the operationalization of GPA, a continuous measure of GPA (GPA percentiles), as well as alternative cutoff points for low GPA (10% and 30%), was used in Additional file G.

To explore how sensitive the results were to the operationalization of graduation years, a continuous (linear) measure of graduation years, as well as an alternative and more fine-grained categorization, was used in Additional file H.

More comprehensive motivations for the supplementary analyses, as well as complete tables and figures, are presented in the respective additional files.

## Results

Table 1 shows results with inpatient treatment for internalizing disorders as the outcome and year 9 GPA as the focal exposure variable, thus addressing RQ1. For the full sample (column 1), the top rows show that the risk of inpatient treatment for internalizing disorders increased from the cohorts graduating in 1990–1997 to the cohorts graduating in 2005–2010 in youths with medium/high GPA (the reference category). The middle rows show that youths with low GPA in the 1990–1997 cohort (the reference category) had a higher risk of being treated for internalizing disorders. The positive (hazard ratio above 1) interaction terms between GPA and graduation cohorts in the bottom rows show that the association between GPA and inpatient treatment for internalizing disorders became stronger in the 2005–2010 graduation cohort. Columns 2–5 show that the association between GPA and inpatient treatment for internalizing disorders became stronger over time in native-born but not immigrant youths, and in girls but not in boys.

**Table 1:** Discrete time proportional hazard models with internalizing disorders as the outcome

	Column 1 Full sample	Column 2 Native-born	Column 3 Immigrant	Column 4 Girls	Column 5 Boys
*Graduation year (ref: 1990–1997)*					
1998–2004	1.797***	1.843***	1.296***	1.827***	1.762***
	[1.726,1.871]	[1.766,1.923]	[1.139,1.474]	[1.740,1.917]	[1.639,1.895]
2005–2010	2.307***	2.382***	1.590***	2.198***	2.623***
	[2.219,2.398]	[2.287,2.481]	[1.390,1.818]	[2.096,2.304]	[2.451,2.807]
*GPA (ref: high/medium GPA)*					
Low GPA	2.214***	2.258***	1.602***	2.626***	2.435***
	[2.105,2.330]	[2.139,2.384]	[1.381,1.857]	[2.459,2.805]	[2.244,2.644]
Graduation year * GPA					
1998–2004 * Low GPA	1.041	1.051	1.114	0.981	1.052
	[0.975,1.110]	[0.981,1.126]	[0.920,1.349]	[0.902,1.066]	[0.948,1.168]
2005–2010* Low GPA	1.118***	1.153***	0.961	1.125**	0.958
	[1.051,1.190]	[1.079,1.231]	[0.789,1.170]	[1.037,1.219]	[0.868,1.058]
N (individuals)	2 252 703	2 077 489	175 158	1 099 819	1 152 850
N (person-years)	17 799 856	16 444 084	1 355 382	8 671 046	9 128 563

Table reports hazard ratios, with 95% confidence intervals in brackets. * *p* < 0.05, ** *p* < 0.01, *** *p* < 0.001. Abbreviations: ref = reference category; GPA = Grade point average

Table 2 shows results with UPS completion as the exposure, thus addressing RQ2. Results are restricted to 3–8 years after graduation from compulsory school. The bottom rows show that the association between UPS completion and inpatient treatment for internalizing disorders became stronger in the 2005–2010 graduation cohort in the full sample, native-born youths and girls, but not in foreign-born youths and boys.

**Table 2:** Discrete time proportional hazard models with internalizing disorders as the outcome

	Column 1 Full sample	Column 2 Native-born	Column 3 Immigrant	Column 4 Girls	Column 5 Boys
*Graduation year (ref: 1990–1997)*					
1998–2004	1.867***	1.918***	1.331***	1.858***	1.881***
	[1.790,1.947]	[1.835,2.005]	[1.165,1.521]	[1.764,1.958]	[1.752,2.019]
2005–2010	2.344***	2.433***	1.515***	2.188***	2.646***
	[2.251,2.441]	[2.331,2.538]	[1.320,1.739]	[2.079,2.302]	[2.474,2.830]
*UPS completion (ref: completed UPS)*					
Not completed UPS	4.226***	4.410***	2.566***	4.314***	4.501***
	[4.006,4.458]	[4.168,4.668]	[2.186,3.012]	[4.032,4.615]	[4.125,4.912]
Graduation year * UPS completion					
1998–2004 * Not completed UPS	0.956	0.961	1.086	0.991	0.926
	[0.893,1.024]	[0.894,1.034]	[0.882,1.337]	[0.908,1.081]	[0.829,1.036]
2005–2010 * Not completed UPS	1.080*	1.097**	1.032	1.200***	0.938
	[1.010,1.154]	[1.023,1.176]	[0.832,1.280]	[1.103,1.306]	[0.843,1.043]
N (individuals)	2 235 735	2 065 463	170 233	1 090 426	1 145 287
N (person-years)	13 100 112	12 117 627	982 312	6 372 269	6 727 750

Table reports hazard ratios, with 95% confidence intervals in brackets. * *p* < 0.05, ** *p* < 0.01, *** *p* < 0.001. Abbreviations: ref = reference category; UPS = Upper secondary school

Table 3 shows the predicted risk of inpatient treatment for internalizing disorders depending on graduation cohort and GPA or UPS completion, with predictions based on the discrete time proportional hazard models. The table furthermore shows the relative risk in low compared to high/medium achieving youths within graduation cohorts, and the equivalent for UPS completion, as well as the change in the relative risk comparing later graduation cohorts with the 1990–1997 cohort. Only results for the full sample is presented for brevity. The relative risk in low-achieving youth increased from 2.213 in the 1990–1997 cohort to 2.471 in the 2005–2010 cohort. Had the relative risk in 2005–2010 stayed constant at its 1990–1997 level, the risk for low-achieving youth in 2005–2010 would have been 0.60% instead of 0.67% (0.0027 × 2.213 = 0.0060), a difference of 0.07% points. By comparison, the average risk in the population as a whole over the period was 0.25% (see Table A1 in Additional file A), meaning that the increase in the excess risk for low-achieving youth corresponds to 28% of the average risk (0.07/0.25 = 0.28). The results with UPS completion as the exposure were similar. The relative risk increased from 3.949 for the 1990–1997 cohort to 4.224 for the 2005–2010 cohort. With stable relative risks, the absolute risk for youth with low attainment (i.e., without completed UPS) in 2005–2010 would have been 0.0109 instead of 0.0116 (before rounding, 0.0028 × 3.949 = 0.0109), a difference of 0.076% points. This corresponds to around 27% of the average risk, which was 0.28% in years 3–8 of follow-up.

**Table 3:** Predicted risks of internalizing disorder by graduation cohort and GPA or UPS completion

	Medium/high GPA	Low GPA			Completed UPS	Not completed UPS		
Graduation year	Predicted risk	Predicted risk	Relative risk	Change in relative risk	Predicted risk	Predicted risk	Relative risk	Change in relative risk
1990–1997	0.0012	0.0026	2.213	Reference	0.0012	0.0047	3.949	Reference
1998–2004	0.0021	0.0048	2.301	1.040	0.0022	0.0083	3.779	0.957
2005–2010	0.0027	0.0067	2.471	1.117	0.0028	0.0116	4.224	1.070

Relative risk = predicted risk for low GPA or not completed UPS divided by predicted risk for medium/high GPA or completed UPS. Change in relative risk = relative risk in later graduation cohorts divided by relative risk in the 1990–1997 graduation cohort

**Fig. 1:**
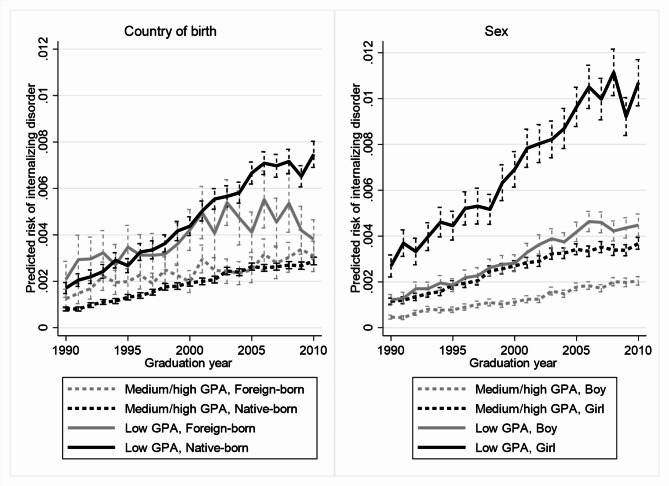
Predicted risks of internalizing disorders by graduation year, GPA, and country of birth or sex. Note: The lines show predicted risks and the vertical spikes 95% confidence intervals. Risks refer to average yearly risks during follow-up

**Fig. 2:**
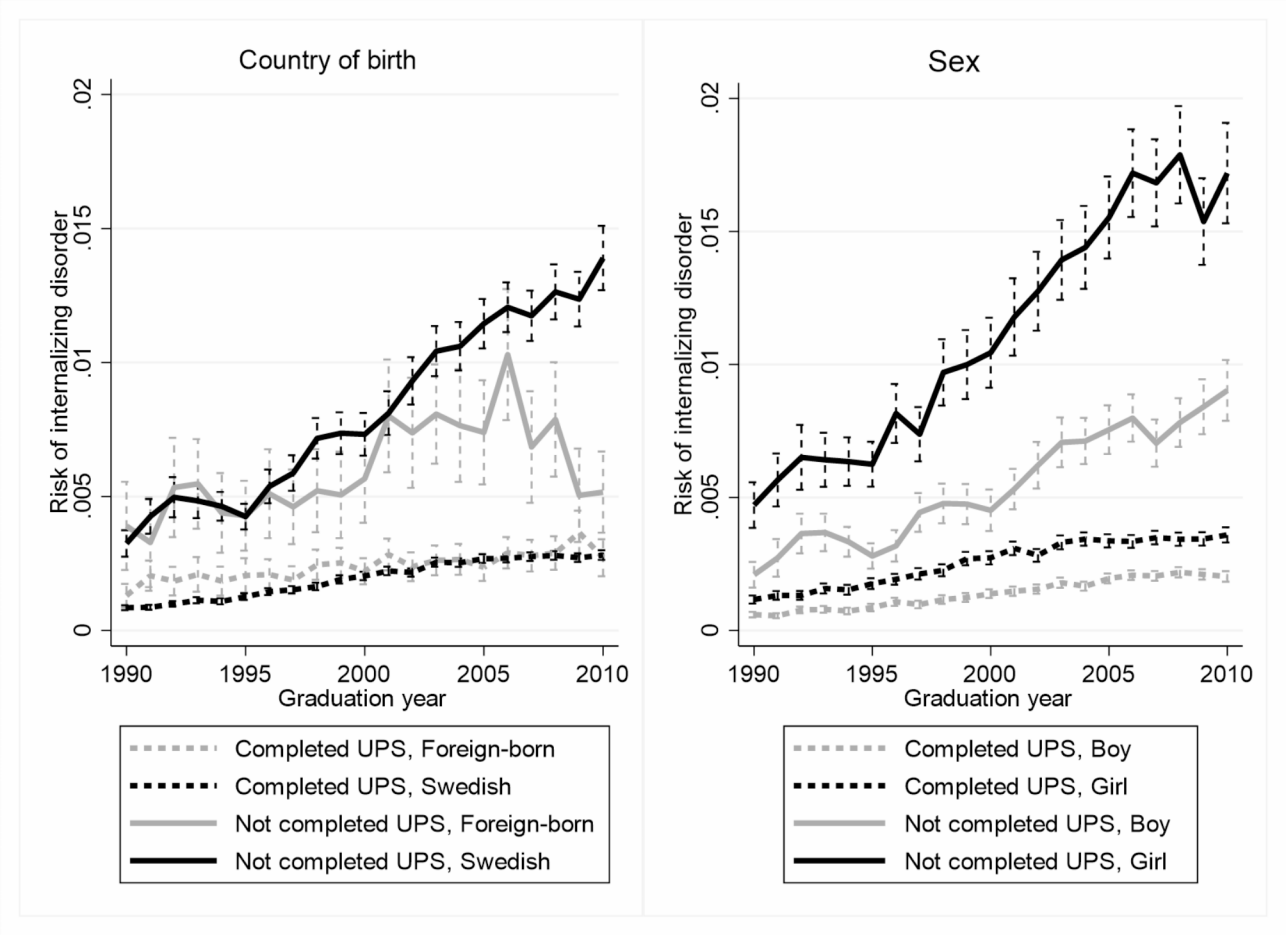
Predicted risks of internalizing disorders by graduation year, UPS completion, and country of birth or sex. Note: The lines show predicted risks and the vertical spikes 95% confidence intervals. Risks refer to average yearly risks during follow-up (3–8 years after compulsory school graduation)

Figures 1 and 2 illustrates the between-group differences in absolute terms, with predictions based on discrete time proportional hazard models equivalent to those used for Table 3, but with one indicator variable for each single graduation year instead of the three broader categories. The predicted risk increased more in the groups with low achievement or attainment (solid lines) than in the groups with medium/high achievement or attainment (dashed lines) among native-born youths, girls and boys, but not foreign-born youths. The predicted risks for native- and foreign-born youths with low achievement or attainment that graduated in the 1990s were largely similar. The risks diverged in later graduation cohorts and were around twice as high for native-born youths with low achievement or attainment graduating in 2010 compared to their foreign-born peers graduating the same year.

### Supplementary and sensitivity analyses

There were no indications that violations of the proportional hazards assumption resulted in biased inferences or misleading conclusions (Supplementary file B). The results of linear probability models (Additional file C); of models with low GPA calculated separately for native-born and immigrant students (Additional file D); of models with UPS completion measured three years after compulsory school graduation (Additional file E); of competing risk models (Additional file F); and of models with alternative operationalizations of GPA (Additional file G) and graduation years (Additional file H) were all substantively similar to the estimates presented in the main manuscript. The competing risk models also showed that the positive association between mortality and, respectively, low GPA and UPS non-completion became stronger in the later graduation cohorts.

## Discussion

The aim of this study was to investigate temporal changes in the association between academic achievement or educational attainment and subsequent inpatient treatment for internalizing disorders among Swedish youths graduating from compulsory school between 1990 and 2010. Two main findings are highlighted.

First, youths with low compulsory school achievement, as well as youths without completed upper secondary school (UPS), had higher risks of being treated for internalizing disorders throughout the period, but this excess risk became stronger in both relative and absolute terms for the later graduation cohorts. These findings are in line with the scarce existing research on the topic. Sörberg-Wallin et al. [10] found that the association between achievement and attempted suicide later in life in Sweden was stronger for the cohort born in 1970 than for the cohort born in 1950. Jablonska et al. [7] compared cohorts that graduated from Swedish compulsory school between 2000 and 2007 and found that the association between achievement and receipt of disability benefits due to mental disorders was stronger for later cohorts. Taken together, these findings provide strong indications that academic achievement and subsequent mental health has become more strongly interrelated in Sweden in recent decades. Studies from other geographical contexts are required to establish whether this is a more widespread pattern.

Second, the increasing excess risk for low-achieving youth and youth without completed UPS were driven by native-born youths and girls. In boys, the excess risk did not change when measured on the relative scale (as hazard ratios), but boys with low achievement and without completed UPS experienced a disproportionate increase in absolute terms. This is because low-achieving boys had higher risks already at the start of the period, and a stable relative risk implies a larger risk difference in absolute terms when both groups experience growing risks over time. The association between inpatient treatment for internalizing disorders and, respectively, low achievement and UPS non-completion, did not change over time for foreign-born youths.

The aim of the study was descriptive – to describe changes in associations over time – and suggested explanations for the observed changes must remain speculative. Nonetheless, the key findings of the study ought to be put in a broader societal and academic context. The first – the growing excess risks for low-achieving youths and youths without completed UPS in the sample as a whole – may potentially be understood either as resulting from stronger social causation over time, or from stronger health selection over time. Stronger social causation implies that the socioeconomic factors that make low achievement or attainment harmful for mental health have become more potent. For instance, the emergence of knowledge economies and the “schooled society” [41] has changed both the instrumental value of education in the labor market, and the moral meaning of education in society at large. Labor market dislocations and growing economic inequality, with fewer low-skilled but well-paid jobs, has resulted in rising returns to education [42], in particular to relative education – that is, one’s educational attainment relative to the educational composition of the labor force [43, 44]. Less educated young workers are increasingly outcompeted from jobs and face deteriorating labor market prospects [45, 46], thus amplifying the negative consequences of low academic achievement. Other sociological theories posit that formal education has increasingly become the primary legitimate means to differentiate people as respectable or successful. This may transform the moral meaning of academic achievement [41] and generate feelings of misrecognition and status loss among the less educated [47, 48].

Growing educational disparities may also come about through stronger health selection over time. As the general education level in the population rises, the less educated may to a disproportionate extent consist of people with internalizing disorders or vulnerability to developing such disorders [49]. Internalizing disorders can also undermine academic achievement [2], and the greater focus on competition and performance in many education systems [50, 51] could disproportionately disadvantage students with such disorders.

Another key finding was the subgroup differences. The growing excess risk of inpatient treatment for internalizing disorders was driven by native-born youths and by girls, while corresponding immigrant youths or boys experienced no or smaller increases. There are large differences in both educational outcomes and mental health or care seeking behaviors between different immigrant groups [31, 52, 53]. The results for immigrant youths can therefore be difficult to interpret due to changes in the size and composition of the immigrant population in Sweden during the period. The finding that the association between educational attainment and treatment for internalizing disorders was generally weaker in immigrant youth is consistent with Nordic research on other mental health outcomes [31]. It is also broadly in line with the “healthy migrant effect” – that migrants are often positively selected in terms of health – found in many Western countries [54]. A tentative explanation for the growing excess risk in native-born youths with low achievement or attainment is, again, either stronger social causation or health selection related to changes in the education system or the way the education system interacts with the labor market.

Previous research has argued that the greater burden of internalizing problems and disorders in girls may be partly due to girls valuing academic achievements higher and being more dependent on educational credentials for their labor market prospects [55, 56]. If the valuation of academic achievement increases in the “schooled society” [41], and if educational attainment becomes more important in knowledge economies, low achievement and attainment may take a greater toll on the mental health of girls. It has also been argued that it is primarily low-achieving and less educated boys or young men that have come out on the losing end of the societal transformations of the last decades [57]. The results of this study do not support this argument: low-achieving and less educated boys in contemporary Sweden certainly have worse mental health than their higher achieving and better educated peers, but was true already in the 1990s.

### Limitations

Although the National Patient Register used in the study generally has high validity [58], the use of specialized inpatient treatment for depression or anxiety disorders as measure of internalizing disorders entails drawbacks. First, internalizing disorders that are not treated in inpatient care are not included. Most youths with internalizing disorders are treated in specialized outpatient care or by general practitioners. Youths treated in inpatient care likely experienced more severe problems and/or greater care needs, and the results may generalize to less severe cases [59]. Access to specialized inpatient care is also better in urban areas. Second, it is not possible to separate trends in symptom prevalence from trends in care-seeking behaviors or treatment practices [60]. Third, there was some underreporting from clinics to the register, especially in the early years of the studied period [61]. It cannot be ruled out that educational differences in care-seeking behaviors, treatment practices or underreporting have changed over the studied period. Fourth, most of the included youths turned 18 during the follow-up period, and treatment guidelines and the main care provider care differ between children aged < 18 and young adults aged ≥ 18. However, there are no shifts in the estimates between the second and the third year after compulsory school graduation, which is typically the years when youths turn 18 (see results in Additional file B). Fifth, although teacher-assigned grades are generally a reliable measure of achievement [62–64], it cannot be ruled out that variation in grading across schools bias the results [25].

## Conclusions

This study investigated changes in the association between academic achievement or educational attainment and subsequent inpatient treatment for internalizing disorders in more than 2 million Swedish compulsory school graduates between 1990 and 2018. The risk of inpatient treatment for internalizing disorders increased disproportionately among both low-achieving youths and youths without completed upper secondary education, and this increase was primarily driven by native-born youths and girls. Future research should investigate whether comparable trends are present in other contexts. Research should also investigate whether low achievement has become more harmful for mental health, for instance because low-achieving students increasingly experience more dire educational and employment prospects (i.e., social causation), or whether youth with internalizing disorders increasingly fail in school (i.e., health selection).

## Electronic supplementary material

Below is the link to the electronic supplementary material.

Supplementary Material 1

## Data Availability

Data are not available for public use. Please contact Umeå SIMSAM Lab for further information on data availability. Replication code will be uploaded upon publication.

## Abbreviations

GPA: Grade point average
UPS: Upper secondary school
ICD: International Classification of Diseases
ISCED: International Standard Classification of Education
