# Supplementary material for: Changes in the association between educational achievement, attainment and subsequent mental health: A survival analysis of 21 Swedish graduation cohorts
Source: BMC Public Health. 2024 Oct 31;24:3016. doi: 10.1186/s12889-024-20554-1 (PMC11526503; doi:10.1186/s12889-024-20554-1)
Supplement: Supplementary file 1 — Supplementary Material 1 [file 12889_2024_20554_MOESM1_ESM.docx]

**Online supplementary materials for *Has academic achievement and educational attainment become more important for subsequent mental health? A survival analysis of 21 Swedish graduation cohorts***

**Additional file A. Descriptive data**

**Additional file B. Investigation of proportional hazards**

**Additional file C. Results from linear probability models**

**Additional file D. GPA categories calculated separately for native-born and immigrant students**

**Additional file E. UPS completion only measured three years after graduation from compulsory school.**

**Additional file F. Competing risk models**

**Additional file G. Alternative operationalizations of GPA**

**Additional file H. Alternative operationalizations of graduation years**

**Additional file A. Descriptive data**

**Table A1. Summary statistics**

| **Graduation cohort** |  | **Internalized disorder** | **Low GPA** | **UPS non-completion** | **Native-born** | **Girl** |
| --- | --- | --- | --- | --- | --- | --- |
| 1990-1997 | Mean | 0.0015 | 0.2130 | 0.1424 | 0.9318 | 0.4875 |
|  | SD | 0.0383 | 0.4094 | 0.3494 | 0.2522 | 0.4998 |
| 1998-2004 | Mean | 0.0027 | 0.2249 | 0.1342 | 0.9096 | 0.4869 |
|  | SD | 0.0520 | 0.4175 | 0.3408 | 0.2867 | 0.4998 |
| 2005-2010 | Mean | 0.0035 | 0.2128 | 0.1233 | 0.9294 | 0.4871 |
|  | SD | 0.0594 | 0.4093 | 0.3288 | 0.2561 | 0.4998 |
| Total | Mean | 0.0025 | 0.2168 | 0.1336 | 0.9239 | 0.4871 |
|  | SD | 0.0503 | 0.4121 | 0.3403 | 0.2652 | 0.4998 |

SD=standard deviation. Note that the proportion with low GPA is slightly greater than 0.2. This is because the quintiles were calculated based on the full sample, including students that graduated more than once from compulsory school, but the latter are not included in the analyses.

**Table A2. Number of observations and events depending on graduation cohort and GPA**

|  | *Medium/high GPA* | | *Low GPA* | |
| --- | --- | --- | --- | --- |
| *Graduation year* | *Observations* | *Events* | *Observations* | *Events* |
| 1990-1997 | 4 965 876 | 5809 | 1 344 106 | 3479 |
| 1998-2004 | 4 462 288 | 9375 | 1 294 947 | 6261 |
| 2005-2010 | 4 512 854 | 12166 | 1 219 785 | 8126 |

Events=Number of years in which a youth was treated for an internalizing disorder.

**Table A3. Number of observations and events depending on graduation cohort and UPS completion**

|  | *Completed UPS* | | *Not completed UPS* | |
| --- | --- | --- | --- | --- |
| *Graduation year* | *Observations* | *Events* | *Observations* | *Events* |
| 1990-1997 | 4 018 952 | 4 748 | 667 240 | 3 113 |
| 1998-2004 | 3 693 713 | 8 135 | 572 661 | 4 766 |
| 2005-2010 | 3 636 062 | 10 005 | 511 484 | 5 946 |

Events=Number of years in which a youth was treated for an internalizing disorder. Note: only included data from years 3-8 after graduation from school year 9.

**Table A4. Proportion of students with low GPA among students that have not completed UPS**

|  | Not completed UPS | Completed UPS |
| --- | --- | --- |
| 1990-1997 | 0.559 | 0.155 |
| 1998-2004 | 0.667 | 0.154 |
| 2005-2010 | 0.670 | 0.149 |

**Figure A1. Average proportion with an internalizing disorder by GPA percentile**

Note: Figure A1 shows the average proportion of youths with an internalizing disorder in each GPA percentile, and for all graduation cohorts combined.

**Additional file B. Investigation of proportional hazards**

Figure B1 and B2 show survival curves depending on GPA or UPS completion, and separately for the three graduation cohorts. The gap between the curves for students with low and medium/high GPA should be proportional across values of follow-up time, and should not cross each other, if the proportional hazards assumption is satisfied. As for GPA (Figure B1), the gap between the curves for students with low and medium/high GPA is, in relative terms, largely proportional across values of follow-up time, and the gaps never cross each other, supporting the proportional hazards assumption. Moreover, Figure B1 also shows that the shape of the curves are similar for the three graduation cohorts, but with a slightly larger gap in the later cohorts, indicating growing differences over time. Note also that the values on the y-axes differ between figures; this is because the overall proportion with internalizing disorders increase for later cohorts. The results for UPS completion in Figure B2 are similar.

Figure B3 and B4 show log-log plots depending on GPA or UPS completion, and separately for the three graduation cohorts. There are no exact ways of determining whether log-log plots indicate support or violations of the proportional hazards assumption, but the lines for students with low and medium/high GPA should run approximately parallel if the assumption is satisfied. As for GPA (Figure B3), the lines are clearly parallel in all graduation cohorts, supporting the proportional hazards assumption. As for UPS completion (Figure B4), the picture is more complex. On the one hand, both the position of the lines and the steepness of the slope of the lines differ depending on UPS completion. On the other hand, the slopes of both lines are positive.

All in all, Figure B1-B4 indicate that the proportional hazards assumption is supported in analyses of GPA, but may be violated in analyses of UPS completion. The most important consequences of violations of the proportional hazards assumption are (1) that estimates of variances of associations, and thus statistical inferences and significance tests, may be biased, and (2) that the average hazard ratio for the full follow-up period may hide substantively important variation in association across follow-up time (Hernán, 2010; Stensrud & Hernán, 2020).

Robust estimates of variances can be obtained using bootstrap methods. Tables B1 and B2 show results using 100 bootstrap replications per model. There are no indications that violations of the proportional hazards assumptions resulted in biased statistical inferences. A more nuanced picture of variations in association across follow-up time can be obtained by stratified the analyses for different periods of follow-up. Tables B3 and and B4 show results for GPA and UPS completion separately for each year of follow-up.

Results in Table B3 indicate no obvious temporal pattern across follow-up time with regard to the changes across graduation cohorts in the association between internalizing disorders and GPA. Although the “main” (in the 1990-1997 cohort) associations between internalizing disorders and GPA were generally strongest in the early years of the follow-up period, there is at most a slight tendency for larger interaction terms in the years closer to compulsory school graduation, but it is weak and not linear. All interaction terms between GPA and the 2005-2010 graduation cohort are positive except for after 5 years, and there are significant interaction terms after 2, 3, 4 and 7 years. Results in Table B4, with UPS completion as focal exposure variable, are similar: the “main” (in the 1990-1997 cohort) associations between internalizing disorders and GPA were generally strongest in the early years of the follow-up period (closer to the typical age of completing upper secondary school). Due to the smaller sample size, estimates for the interactions between cohort and UPS non-completion are not significant in any of the single follow-up years. The point estimates for the interaction terms are larger in later follow-up years, indicating that the association may grow stronger over time.

In sum, Tables B1-B4 suggest that potential violations of the proportional hazards assumption do not result in biased inferences or misleading conclusions regarding cross-cohort changes in the associations between internalizing disorders and GPA or UPS completion.

**Figure B1. Survival curves, by graduation cohort and GPA**

**Figure B2. Survival curves, by graduation cohort and UPS completion**

**Figure B3. Log-log plots, by graduation cohort and GPA**

**Figure B4. Log-log plots, by graduation cohort and UPS completion**

**Table B1 Discrete time proportional hazard models with internalizing disorders as the outcome.**

|  | Column 1  Full sample | Column 2  Native-born | Column 3  Immigrant | Column 4  Girls | Column 5  Boys |
| --- | --- | --- | --- | --- | --- |
| *Graduation year (ref: 1990-1997)* |  |  |  |  |  |
| 1998-2004 | 1.797*** | 1.843*** | 1.296*** | 1.827*** | 1.762*** |
|  | [1.736,1.862] | [1.763,1.930] | [1.150,1.462] | [1.741,1.918] | [1.639,1.897] |
| 2005-2010 | 2.307*** | 2.382*** | 1.590*** | 2.198*** | 2.623*** |
|  | [2.218,2.404] | [2.305,2.466] | [1.387,1.824] | [2.111,2.293] | [2.440,2.824] |
| *GPA (ref: high/medium GPA)* |  |  |  |  |  |
| Low GPA | 2.214*** | 2.258*** | 1.602*** | 2.626*** | 2.435*** |
|  | [2.109,2.329] | [2.148,2.378] | [1.395,1.840] | [2.460,2.811] | [2.268,2.618] |
| *Graduation year * GPA* |  |  |  |  |  |
| 1998-2004 * Low GPA | 1.041 | 1.051 | 1.114 | 0.981 | 1.052 |
|  | [0.980,1.107] | [0.983,1.126] | [0.917,1.356] | [0.903,1.067] | [0.951,1.166] |
| 2005-2010* Low GPA | 1.118*** | 1.153*** | 0.961 | 1.125** | 0.958 |
|  | [1.065,1.177] | [1.086,1.227] | [0.800,1.155] | [1.035,1.227] | [0.873,1.054] |
| N (students) | 2 252 703 | 2 077 489 | 175 158 | 1 099 819 | 1 152 850 |
| N (person-years) | 17 799 856 | 16 444 084 | 1 355 382 | 8 671 046 | 9 128 563 |

Table reports hazard ratios, with 95% bootstrap confidence intervals in brackets. * p<0.05, ** p<0.01, *** p<0.001. Abbreviations: ref = reference category; GPA = Grade point average.

**Table B2.** **Discrete time proportional hazard models with internalizing disorders as the outcome.**

|  | Column 1  Full sample | Column 2  Native-born | Column 3  Immigrant | Column 4  Girls | Column 5  Boys |
| --- | --- | --- | --- | --- | --- |
| *Graduation year (ref: 1990-1997)* |  |  |  |  |  |
| 1998-2004 | 1.868*** | 1.919*** | 1.332*** | 1.860*** | 1.881*** |
|  | [1.787,1.952] | [1.846,1.996] | [1.167,1.519] | [1.781,1.942] | [1.759,2.012] |
| 2005-2010 | 2.346*** | 2.435*** | 1.516*** | 2.190*** | 2.648*** |
|  | [2.248,2.447] | [2.340,2.533] | [1.328,1.731] | [2.089,2.296] | [2.501,2.803] |
| *UPS completion (ref: completed UPS)* |  |  |  |  |  |
| Not completed UPS | 4.235*** | 4.420*** | 2.570*** | 4.326*** | 4.508*** |
|  | [3.995,4.489] | [4.186,4.666] | [2.188,3.018] | [4.073,4.595] | [4.182,4.860] |
| *Graduation year * UPS completion* |  |  |  |  |  |
| 1998-2004 * Not completed UPS | 0.958 | 0.963 | 1.087 | 0.993 | 0.927 |
|  | [0.884,1.037] | [0.893,1.038] | [0.898,1.316] | [0.911,1.082] | [0.844,1.018] |
| 2005-2010* Not completed UPS | 1.083* | 1.100** | 1.033 | 1.206*** | 0.940 |
|  | [1.005,1.167] | [1.036,1.169] | [0.827,1.290] | [1.112,1.307] | [0.853,1.035] |
| N (students) | 2 235 735 | 2 065 463 | 170 233 | 1 090 426 | 1 145 287 |
| N (person-years) | 13 100 112 | 12 117 627 | 982 312 | 6 372 269 | 6 727 750 |

Table reports hazard ratios, with 95% bootstrap confidence intervals in brackets. * p<0.05, ** p<0.01, *** p<0.001. Abbreviations: ref = reference category; UPS = Upper secondary school.

**Table B3. Discrete time proportional hazard models with internalizing disorders as the outcome. Separate models for each year of follow-up.**

|  | Year 1 | Year 2 | Year 3 | Year 4 | Year 5 | Year 6 | Year 7 | Year 8 |
| --- | --- | --- | --- | --- | --- | --- | --- | --- |
| *Graduation year (ref: 1990-1997)* |  |  |  |  |  |  |  |  |
| 1998-2004 | 2.012*** | 2.048*** | 1.842*** | 1.782*** | 1.808*** | 1.794*** | 1.693*** | 1.681*** |
|  | [1.760,2.301] | [1.834,2.287] | [1.671,2.031] | [1.628,1.951] | [1.655,1.975] | [1.647,1.953] | [1.559,1.838] | [1.551,1.822] |
| 2005-2010 | 2.962*** | 2.872*** | 2.624*** | 2.411*** | 2.361*** | 2.199*** | 1.971*** | 1.896*** |
|  | [2.612,3.359] | [2.587,3.189] | [2.394,2.876] | [2.213,2.627] | [2.170,2.568] | [2.026,2.388] | [1.819,2.135] | [1.753,2.051] |
| *GPA (ref: high/medium GPA)* |  |  |  |  |  |  |  |  |
| Low GPA | 2.627*** | 2.256*** | 2.097*** | 2.050*** | 2.476*** | 2.235*** | 2.152*** | 2.101*** |
|  | [2.224,3.104] | [1.952,2.606] | [1.845,2.383] | [1.820,2.309] | [2.217,2.764] | [2.004,2.493] | [1.936,2.392] | [1.894,2.331] |
| *Graduation year * GPA* |  |  |  |  |  |  |  |  |
| 1998-2004 * Low GPA | 1.053 | 0.990 | 1.090 | 1.163* | 0.887 | 1.074 | 1.059 | 1.027 |
|  | [0.858,1.293] | [0.828,1.183] | [0.929,1.278] | [1.003,1.349] | [0.771,1.022] | [0.936,1.231] | [0.926,1.211] | [0.899,1.172] |
| 2005-2010* Low GPA | 1.130 | 1.190* | 1.259** | 1.176* | 0.925 | 1.072 | 1.140* | 1.095 |
|  | [0.931,1.371] | [1.006,1.407] | [1.084,1.463] | [1.021,1.355] | [0.808,1.058] | [0.938,1.224] | [1.001,1.299] | [0.962,1.246] |
| N (students) | 2 252 703 | 2 247 893 | 2 245 311 | 2 236 260 | 2 224 314 | 2 210 957 | 2 197 765 | 2 184 653 |
| N (person-years) | 2 252 703 | 2 247 893 | 2 245 311 | 2 236 260 | 2 224 314 | 2 210 957 | 2 197 765 | 2 184 653 |

Table reports hazard ratios, with 95% confidence intervals in brackets. * p<0.05, ** p<0.01, *** p<0.001. Abbreviations: ref = reference category; GPA = Grade point average.

**Table B4. Discrete time proportional hazard models with internalizing disorders as the outcome. Separate models for each year of follow-up.**

|  | Year 3 | Year 4 | Year 5 | Year 6 | Year 7 | Year 8 |
| --- | --- | --- | --- | --- | --- | --- |
| *Graduation year (ref: 1990-1997)* |  |  |  |  |  |  |
| 1998-2004 | 2.117*** | 1.947*** | 1.873*** | 1.928*** | 1.781*** | 1.768*** |
|  | [1.862,2.408] | [1.766,2.146] | [1.715,2.046] | [1.775,2.093] | [1.647,1.925] | [1.639,1.908] |
| 2005-2010 | 3.118*** | 2.712*** | 2.447*** | 2.313*** | 2.109*** | 2.046*** |
|  | [2.762,3.520] | [2.473,2.974] | [2.249,2.662] | [2.136,2.505] | [1.955,2.274] | [1.896,2.208] |
| *UPS completion (ref: completed UPS)* |  |  |  |  |  |  |
| Not completed UPS | 5.711*** | 4.978*** | 4.711*** | 4.010*** | 3.621*** | 3.656*** |
|  | [5.017,6.502] | [4.437,5.584] | [4.220,5.260] | [3.587,4.483] | [3.242,4.043] | [3.276,4.079] |
| *Graduation year * UPS completion* |  |  |  |  |  |  |
| 1998-2004 * Not completed UPS | 0.897 | 1.005 | 0.874 | 0.938 | 0.968 | 0.925 |
|  | [0.763,1.053] | [0.870,1.161] | [0.758,1.007] | [0.814,1.081] | [0.840,1.115] | [0.802,1.066] |
| 2005-2010* Not completed UPS | 0.959 | 0.981 | 0.952 | 1.083 | 1.086 | 1.004 |
|  | [0.824,1.116] | [0.855,1.126] | [0.832,1.090] | [0.944,1.242] | [0.945,1.246] | [0.870,1.160] |
| N (students) | 2 229 331 | 2 221 302 | 2 209 382 | 2 196 756 | 2 183 795 | 2 059 546 |
| N (person-years) | 2 229 331 | 2 221 302 | 2 209 382 | 2 196 756 | 2 183 795 | 2 059 546 |

Table reports hazard ratios, with 95% confidence intervals in brackets. * p<0.05, ** p<0.01, *** p<0.001. Abbreviations: ref = reference category; UPS = Upper secondary school.

**Additional file C. Results from linear probability models**

Comparisons of results from different nonlinear probability models or limited dependent variable models – including logistic, probit and, importantly in this context, complementary log-log models – can reflect differences in unobserved heterogeneity across models in addition to differential effects of the included variables (Breen et al., 2018; Mood, 2010). This is because these models cannot separately identify coefficients and the residual variation of the dependent variable. This, in turn, means that differences in the coefficients for the same independent variable across different models – either models with the same variables but fitted to different samples (e.g., subgroups), or models fitted on the same sample but with different covariates included – can reflect either “real” differences in the underlying association between the focal independent variable and the outcome (net of covariates), or differences in the residual variances between the models. In general, and all else being equal, if the residual variance is smaller, the coefficients will be larger.

This has implications for the interpretation of the results of the present study, since coefficients for the same variables (graduation year and GPA) are compared across models fitted on different samples or subgroups (defined by sex and migration background). It is not known if the residual variance differs between these groups, and hence if the differences between the subgroups reflect “real” differences in the underlying association between internalizing disorders and, respectively, graduation year and GPA.

One solution to this is to estimate the models using linear (ordinary least squares) regression, that is, linear probability models, the results of which are not sensitive to differences in unobserved heterogeneity across models (Breen et al., 2018; Mood, 2010). If the same pattern of results holds for the linear probability models as for the complementary log-log models, we can with greater confidence rule out the possibility that the results are driven by differences in residual variance across the subgroups. Unlike the complementary log-log models, linear regression assumes linear relationships between independent variables and the outcome. However, in all independent variables are entered as categorical variables, meaning that potential non-linearities are not an issue.

Tables C1-C2 shows that the results are qualitatively similar to the main results presented in the paper: the increase across cohorts in the association between internalizing disorders and, respectively, GPA and UPS completion is stronger for native-born students and girls.

**Table C1 – Linear regression models with internalizing disorders as the outcome.**

|  | Column 1  Full sample | Column 2  Native-born | Column 3  Immigrant | Column 4  Girls | Column 5  Boys |
| --- | --- | --- | --- | --- | --- |
| *Graduation year (ref: 1990-1997)* |  |  |  |  |  |
| 1998-2004 | 0.000931*** | 0.000949*** | 0.000552*** | 0.00130*** | 0.000551*** |
|  | (0.0000330) | (0.0000340) | (0.000139) | (0.0000540) | (0.0000362) |
| 2005-2010 | 0.00153*** | 0.00155*** | 0.00110*** | 0.00188*** | 0.00117*** |
|  | (0.0000364) | (0.0000372) | (0.000167) | (0.0000585) | (0.0000418) |
| *GPA (ref: high/medium GPA)* |  |  |  |  |  |
| Low GPA | 0.00142*** | 0.00142*** | 0.00112*** | 0.00255*** | 0.00104*** |
|  | (0.0000559) | (0.0000586) | (0.000192) | (0.000117) | (0.0000571) |
| *Graduation year * GPA* |  |  |  |  |  |
| 1998-2004 * Low GPA | 0.00132*** | 0.00143*** | 0.000773** | 0.00196*** | 0.000950*** |
|  | (0.0000982) | (0.000105) | (0.000291) | (0.000199) | (0.000100) |
| 2005-2010* Low GPA | 0.00255*** | 0.00287*** | 0.000474 | 0.00416*** | 0.00148*** |
|  | (0.000114) | (0.000123) | (0.000321) | (0.000231) | (0.000115) |
| N (students) | 2 252 703 | 2 077 489 | 175 158 | 1 099 819 | 1 152 850 |
| N (person-years) | 17 799 856 | 16 444 084 | 1 355 382 | 8 671 046 | 9 128 563 |

Table reports linear regression coefficients, with standard errors in parentheses. * p<0.05, ** p<0.01, *** p<0.001. Abbreviations: ref = reference category; GPA = Grade point average.

**Table C2. Linear regression models with internalizing disorders as the outcome**

|  | Column 1  Full sample | Column 2  Native-born | Column 3  Immigrant | Column 4  Girls | Column 5  Boys |
| --- | --- | --- | --- | --- | --- |
| *Graduation year (ref: 1990-1997)* |  |  |  |  |  |
| 1998-2004 | 0.00102*** | 0.00104*** | 0.000629*** | 0.00136*** | 0.000697*** |
|  | (0.0000353) | (0.0000364) | (0.000147) | (0.0000583) | (0.0000401) |
| 2005-2010 | 0.00159*** | 0.00162*** | 0.000979*** | 0.00188*** | 0.00130*** |
|  | (0.0000384) | (0.0000395) | (0.000166) | (0.0000623) | (0.0000454) |
| *UPS completion (ref: completed UPS)* |  |  |  |  |  |
| Not completed UPS | 0.00369*** | 0.00374*** | 0.00290*** | 0.00508*** | 0.00266*** |
|  | (0.000103) | (0.000110) | (0.000305) | (0.000182) | (0.000114) |
| *Graduation year * UPS completion* |  |  |  |  |  |
| 1998-2004 * Not completed UPS | 0.00263*** | 0.00289*** | 0.00142** | 0.00403*** | 0.00172*** |
|  | (0.000182) | (0.000197) | (0.000478) | (0.000329) | (0.000197) |
| 2005-2010* Not completed UPS | 0.00541*** | 0.00601*** | 0.00155** | 0.00837*** | 0.00340*** |
|  | (0.000217) | (0.000236) | (0.000529) | (0.000398) | (0.000232) |
| N (students) | 2 235 735 | 2 065 463 | 170 233 | 1 090 426 | 1 145 287 |
| N (person-years) | 13 100 112 | 12 117 627 | 982 312 | 6 372 269 | 6 727 750 |

Table reports linear regression coefficients, with standard errors in parentheses. * p<0.05, ** p<0.01, *** p<0.001. Abbreviations: ref = reference category; UPS = Upper secondary school.

**Additional file D. GPA categories calculated separately for native-born and immigrant students**

The composition of the group with low GPA may have changed across graduation cohorts due to immigration, with relatively more immigrant students among those with low grades in more recent cohorts. If so, changes in the association between GPA and internalizing disorders across cohorts may partly be due to compositional changes. Since the aim of the present study is descriptive, this would not be a source of bias, but it would influence the interpretation of the results. Table 1D therefore shows results of analyses in which the low GPA category was calculated separately for native-born and immigrant students. The results are similar to the main results reported in the paper.

**Table D1. Discrete time proportional hazard models with internalizing disorders as the outcome**

|  | Column 2  Native-born | Column 3  Immigrant |
| --- | --- | --- |
| *Graduation year (ref: 1990-1997)* |  |  |
| 1998-2004 | 1.847*** | 1.342*** |
|  | [1.770,1.928] | [1.197,1.506] |
| 2005-2010 | 2.350*** | 1.713*** |
|  | [2.255,2.449] | [1.524,1.927] |
| *GPA (ref: high/medium GPA)* |  |  |
| Low GPA | 2.245*** | 1.669*** |
|  | [2.127,2.369] | [1.425,1.954] |
| *Graduation year * GPA* |  |  |
| 1998-2004 * Low GPA | 1.058 | 1.138 |
|  | [0.987,1.133] | [0.927,1.398] |
| 2005-2010* Low GPA | 1.119*** | 0.823 |
|  | [1.048,1.194] | [0.663,1.021] |
| N (students) | 2 077 489 | 175 158 |
| N (person-years) | 16 444 084 | 1 355 382 |

Table reports hazard ratios, with 95% confidence intervals in brackets. * p<0.05, ** p<0.01, *** p<0.001. Abbreviations: ref = reference category; GPA = Grade point average.

**Additional file E. UPS completion only measured three years after graduation from compulsory school.**

Unlike year 9 GPA, UPS completion is time-varying variable. Thus, the results related to UPS completion may be due to reverse causation, that is, internalizing disorders causing or impeding changes in UPS completion. Since the present study is descriptive, this is not necessarily a problem, but it may influence the interpretation of the results.

Table E1 therefore shows results where UPS completion is measured only three years after graduation from compulsory school. That is, students that completed upper secondary school at most three years after graduating from compulsory school are coded 0, and students that had not completed upper secondary school three years after graduating from compulsory school are coded 1. Upper secondary school follows directly from compulsory school and lasts for three years, and three years after graduation from compulsory school is thus the typical time that students attain upper secondary credentials. The results using this alternative definition of UPS completion are largely similar to the main results presented in the paper.

**Table E1. Discrete time proportional hazard models with internalizing disorders as the outcome**

|  | Column 1  Full sample | Column 2  Native-born | Column 3  Immigrant | Column 4  Girls | Column 5  Boys |
| --- | --- | --- | --- | --- | --- |
| *Graduation year (ref: 1990-1997)* |  |  |  |  |  |
| 1998-2004 | 1.829*** | 1.883*** | 1.251** | 1.841*** | 1.804*** |
|  | [1.742,1.921] | [1.789,1.982] | [1.069,1.465] | [1.733,1.955] | [1.661,1.960] |
| 2005-2010 | 2.320*** | 2.408*** | 1.479*** | 2.180*** | 2.589*** |
|  | [2.214,2.432] | [2.293,2.530] | [1.257,1.741] | [2.056,2.313] | [2.395,2.798] |
| *UPS completion (ref: completed UPS)* |  |  |  |  |  |
| Not completed UPS | 3.714*** | 3.864*** | 2.231*** | 3.870*** | 3.751*** |
|  | [3.523,3.916] | [3.654,4.086] | [1.898,2.624] | [3.621,4.135] | [3.438,4.092] |
| *Graduation year * UPS completion* |  |  |  |  |  |
| 1998-2004 * Not completed UPS | 1.015 | 1.016 | 1.193 | 1.023 | 1.023 |
|  | [0.948,1.086] | [0.946,1.091] | [0.968,1.470] | [0.939,1.113] | [0.916,1.144] |
| 2005-2010* Not completed UPS | 1.067* | 1.083* | 1.042 | 1.133** | 0.983 |
|  | [1.000,1.139] | [1.011,1.159] | [0.840,1.293] | [1.043,1.230] | [0.884,1.092] |
| N (students) | 2 229 331 | 2 061 005 | 168 287 | 1 087 584 | 1 141 725 |
| N (person-years) | 13 243 577 | 12 251 961 | 991 396 | 6 447 715 | 6 795 730 |

Table reports hazard ratios, with 95% confidence intervals in brackets. * p<0.05, ** p<0.01, *** p<0.001. Abbreviations: ref = reference category; UPS = Upper secondary school.

**Additional file F. Competing risks models.**

In the main analyses, participants that die during follow-up are treated as censored. Another approach is to view mortality as a competing event, or risk, that prevents the main event of interest, that is, treatment for internalizing disorders. Additional file F present results from competing risk models with a multinomial outcome variable that takes the value 1 if the participant is treated for an internalizing disorder in a given year, 2 if the participant dies in a given year, and 0 if neither of these events takes place and the participant is not censored due to migration or reaching the end of follow up. By estimating effects on all outcome simultaneously, competing risk models enable the disentangling of the effects of the covariates on the event of interest (treatment for internalizing disorders) from their effects of competing events (mortality). Discrete time multinomial models for competing risks are also more efficient than models where compering events are treated as censored (Allison, 1982). Considering mortality as an outcome may also be seen to be of substantive interest since estimates of temporal trends in all-cause mortality, unlike comparable estimates for internalizing disorders, are not influenced by changes in diagnostic practices, healthcare seeking behaviors or similar factors. It should be noted that data on UPS completion are in most cases not available in the year that a participant dies. For this reason, UPS completion has been lagged one year in the models presented in Table F2, meaning that these results for not fully comparable to the main results presented in the paper. It should also be noted that data on specific causes of death are not available.

Tables F1-F2 shows that the results with internalizing disorders as the outcome are very similar to the main results presented in the paper, suggesting that competing risks due to death do not lead to bias. As for results with mortality as the outcome, it is notable that the positive association between mortality and low GPA became gradually stronger in the later graduation cohorts. The increase in the association is significant for native-born bot not immigrant youth, and it is somewhat larger for girls compared to boys. The positive association between mortality and UPS non-completion only became stronger in the 2005-2010 graduation cohorts, and only for native-born youth and for girls.

**Table F1. Discrete time competing risk models with internalizing disorders or death as outcomes**

|  | Column 1  Full sample | Column 2  Native-born | Column 3  Immigrant | Column 4  Girls | Column 5  Boys |
| --- | --- | --- | --- | --- | --- |
| *Outcome 1: Internalizing disorders* |  |  |  |  |  |
| *Graduation year (ref: 1990-1997)* |  |  |  |  |  |
| 1998-2004 | 1.798*** | 1.844*** | 1.296*** | 1.828*** | 1.763*** |
|  | [1.727,1.872] | [1.767,1.924] | [1.139,1.475] | [1.741,1.919] | [1.639,1.896] |
| 2005-2010 | 2.308*** | 2.384*** | 1.590*** | 2.200*** | 2.624*** |
|  | [2.221,2.400] | [2.289,2.483] | [1.390,1.819] | [2.098,2.306] | [2.452,2.809] |
| *GPA (ref: high/medium GPA)* |  |  |  |  |  |
| Low GPA | 2.217*** | 2.261*** | 1.603*** | 2.630*** | 2.438*** |
|  | [2.107,2.333] | [2.142,2.387] | [1.382,1.859] | [2.462,2.809] | [2.246,2.647] |
| *Graduation year * GPA* |  |  |  |  |  |
| 1998-2004 * Low GPA | 1.041 | 1.052 | 1.115 | 0.982 | 1.053 |
|  | [0.976,1.111] | [0.982,1.127] | [0.921,1.350] | [0.903,1.067] | [0.948,1.169] |
| 2005-2010 * Low GPA | 1.120*** | 1.154*** | 0.961 | 1.127** | 0.959 |
|  | [1.052,1.192] | [1.081,1.233] | [0.789,1.171] | [1.040,1.222] | [0.869,1.060] |
| *Outcome 2: Death* |  |  |  |  |  |
| *Graduation year (ref: 1990-1997)* |  |  |  |  |  |
| 1998-2004 | 0.912* | 0.938 | 0.631*** | 0.875* | 0.922 |
|  | [0.847,0.983] | [0.868,1.014] | [0.484,0.822] | [0.765,0.999] | [0.843,1.008] |
| 2005-2010 | 0.773*** | 0.792*** | 0.565*** | 0.763*** | 0.767*** |
|  | [0.715,0.835] | [0.731,0.858] | [0.418,0.763] | [0.664,0.876] | [0.698,0.842] |
| *GPA (ref: high/medium GPA)* |  |  |  |  |  |
| Low GPA | 2.444*** | 2.508*** | 1.727*** | 2.076*** | 2.154*** |
|  | [2.258,2.645] | [2.307,2.725] | [1.336,2.231] | [1.753,2.458] | [1.966,2.359] |
| *Graduation year * GPA* |  |  |  |  |  |
| 1998-2004 * Low GPA | 1.125* | 1.130* | 1.295 | 1.280* | 1.121 |
|  | [1.003,1.261] | [1.001,1.274] | [0.891,1.883] | [1.010,1.622] | [0.982,1.280] |
| 2005-2010* Low GPA | 1.267*** | 1.300*** | 1.121 | 1.389** | 1.298*** |
|  | [1.126,1.427] | [1.149,1.472] | [0.735,1.711] | [1.088,1.772] | [1.131,1.490] |
| N (students) | 2 252 703 | 2 077 489 | 175 158 | 1 099 819 | 1 152 850 |
| N (person-years) | 17 799 856 | 16 444 084 | 1 355 382 | 8 671 046 | 9 128 563 |

Table reports hazard ratios, with 95% confidence intervals in brackets. * p<0.05, ** p<0.01, *** p<0.001. Abbreviations: ref = reference category; GPA = Grade point average

**Table F2. Discrete time competing risk models with internalizing disorders or death as outcomes**

|  | Column 1  Full sample | Column 2  Native-born | Column 3  Immigrant | Column 4  Girls | Column 5  Boys |
| --- | --- | --- | --- | --- | --- |
| *Outcome 1: Internalizing disorders* |  |  |  |  |  |
| *Graduation year (ref: 1990-1997)* |  |  |  |  |  |
| 1998-2004 | 1.829*** | 1.880*** | 1.297*** | 1.800*** | 1.880*** |
|  | [1.750,1.912] | [1.795,1.970] | [1.125,1.494] | [1.703,1.901] | [1.746,2.025] |
| 2005-2010 | 2.239*** | 2.323*** | 1.442*** | 2.081*** | 2.534*** |
|  | [2.146,2.336] | [2.222,2.429] | [1.247,1.668] | [1.973,2.195] | [2.363,2.718] |
| *UPS completion (ref: completed UPS)* |  |  |  |  |  |
| Not completed UPS | 3.799*** | 3.968*** | 2.352*** | 3.810*** | 4.136*** |
|  | [3.605,4.003] | [3.755,4.194] | [2.007,2.757] | [3.567,4.070] | [3.796,4.508] |
| *Graduation year * UPS completion* |  |  |  |  |  |
| 1998-2004 * Not completed UPS | 0.972 | 0.972 | 1.105 | 1.028 | 0.903 |
|  | [0.911,1.037] | [0.908,1.041] | [0.904,1.350] | [0.948,1.115] | [0.811,1.006] |
| 2005-2010* Not completed UPS | 1.114*** | 1.127*** | 1.105 | 1.221*** | 0.983 |
|  | [1.047,1.186] | [1.056,1.203] | [0.901,1.355] | [1.129,1.322] | [0.888,1.089] |
| *Outcome 2: Death* |  |  |  |  |  |
| *Graduation year (ref: 1990-1997)* |  |  |  |  |  |
| 1998-2004 | 0.954 | 0.987 | 0.617** | 0.929 | 0.965 |
|  | [0.878,1.036] | [0.906,1.076] | [0.459,0.829] | [0.788,1.096] | [0.877,1.062] |
| 2005-2010 | 0.805*** | 0.823*** | 0.607** | 0.831* | 0.798*** |
|  | [0.738,0.878] | [0.752,0.901] | [0.441,0.836] | [0.701,0.985] | [0.721,0.883] |
| *UPS completion (ref: completed UPS)* |  |  |  |  |  |
| Not completed UPS | 3.108*** | 3.224*** | 2.083*** | 3.026*** | 2.948*** |
|  | [2.834,3.409] | [2.927,3.552] | [1.528,2.840] | [2.514,3.642] | [2.650,3.279] |
| *Graduation year * UPS completion* |  |  |  |  |  |
| 1998-2004 * Not completed UPS | 1.096 | 1.081 | 1.469 | 1.214 | 1.043 |
|  | [0.966,1.244] | [0.946,1.234] | [0.963,2.241] | [0.944,1.563] | [0.901,1.207] |
| 2005-2010* Not completed UPS | 1.213** | 1.229** | 1.130 | 1.157 | 1.207* |
|  | [1.063,1.384] | [1.072,1.411] | [0.705,1.809] | [0.888,1.507] | [1.037,1.405] |
| N (students) | 2243653 | 2071984 | 171626 | 1095494 | 1148134 |
| N (person-years) | 13 264 598 | 12 267 659 | 996 741 | 6 458 083 | 6 806 406 |

Table reports hazard ratios, with 95% confidence intervals in brackets. * p<0.05, ** p<0.01, *** p<0.001. Abbreviations: ref = reference category; UPS = Upper secondary school.

**Additional file G. Alternative operationalizations of GPA**

Additional file G investigates alternative ways of measuring GPA. Table G1 shows results from logistic regression models with internalizing disorders as the outcome and with GPA percentiles measured as a continuous variable (range 1-100) as the focal independent variable. The models do not include polynomial terms and thus assumes that the log odds of internalizing disorders are a linear function of the GPA percentile scores. Table G2 shows results from equivalent models but with GPA percentiles dichotomized at the 10^th^ percentile score, meaning that low GPA corresponds to the lowest 10% and medium/high GPA to the highest 90%. Table G3 shows results from equivalent models but with GPA percentiles dichotomized at the 30^th^ percentile score, meaning that low GPA corresponds to the lowest 30% and medium/high GPA to the highest 70%.

The results with continuous GPA show that higher GPA is linearly associated with a lower risk of internalizing disorders, and that this linear association became stronger in the 2005-2010 graduation cohort; a result driven by native-born youth and girls. The results using a 10% cutoff for low GPA are similar to, but somewhat stronger than, the main results, as can be seen from the larger interaction terms. The results using a 30% cutoff for low GPA are similar to, but somewhat weaker than, the main results, as can be seen from the smaller interaction terms. This suggest that the main results are driven by the very lowest achieving youth.

**Table G1 – Discrete time proportional hazard models with internalizing disorders as the outcome. Continuous measure of GPA.**

|  | Column 1  Full sample | Column 2  Native-born | Column 3  Immigrant | Column 4  Girls | Column 5  Boys |
| --- | --- | --- | --- | --- | --- |
| *Graduation year (ref: 1990-1997)* |  |  |  |  |  |
| 1998-2004 | 1.912*** | 2.005*** | 1.381*** | 1.860*** | 1.955*** |
|  | [1.807,2.022] | [1.887,2.130] | [1.184,1.610] | [1.730,2.000] | [1.786,2.140] |
| 2005-2010 | 2.634*** | 2.845*** | 1.434*** | 2.553*** | 2.569*** |
|  | [2.495,2.780] | [2.685,3.015] | [1.229,1.672] | [2.380,2.739] | [2.358,2.799] |
| *GPA* |  |  |  |  |  |
| GPA percentile | 0.986*** | 0.986*** | 0.989*** | 0.983*** | 0.983*** |
|  | [0.985,0.987] | [0.985,0.987] | [0.986,0.992] | [0.982,0.985] | [0.981,0.985] |
| *Graduation year * GPA* |  |  |  |  |  |
| 1998-2004 * GPA percentile | 0.999 | 0.999 | 1.000 | 1.000 | 0.998 |
|  | [0.998,1.001] | [0.998,1.000] | [0.996,1.004] | [0.999,1.002] | [0.996,1.000] |
| 2005-2010* GPA percentile | 0.998*** | 0.997*** | 1.003 | 0.998** | 1.000 |
|  | [0.997,0.999] | [0.996,0.998] | [0.999,1.007] | [0.997,0.999] | [0.998,1.002] |
| N (students) | 2 252 703 | 2 077 489 | 175 158 | 1 099 819 | 1 152 850 |
| N (person-years) | 17 799 856 | 16 444 084 | 1 355 382 | 8 671 046 | 9 128 563 |

Table reports hazard ratios, with 95% confidence intervals in brackets. * p<0.05, ** p<0.01, *** p<0.001. Abbreviations: ref = reference category; GPA = Grade point average

**Table G2 – Discrete time proportional hazard models with internalizing disorders as the outcome. 10 % as cutoff for low GPA.**

|  | Column 1  Full sample | Column 2  Native-born | Column 3  Immigrant | Column 4  Girls | Column 5  Boys |
| --- | --- | --- | --- | --- | --- |
| *Graduation year (ref: 1990-1997)* |  |  |  |  |  |
| 1998-2004 | 1.843*** | 1.895*** | 1.328*** | 1.859*** | 1.858*** |
|  | [1.778,1.912] | [1.824,1.969] | [1.186,1.486] | [1.779,1.944] | [1.745,1.979] |
| 2005-2010 | 2.360*** | 2.439*** | 1.648*** | 2.240*** | 2.687*** |
|  | [2.279,2.444] | [2.351,2.530] | [1.464,1.854] | [2.145,2.339] | [2.532,2.852] |
| *GPA (ref: high/medium GPA)* |  |  |  |  |  |
| Low GPA | 2.480*** | 2.545*** | 1.746*** | 2.911*** | 2.788*** |
|  | [2.342,2.626] | [2.395,2.706] | [1.482,2.059] | [2.695,3.145] | [2.555,3.042] |
| *Graduation year * GPA* |  |  |  |  |  |
| 1998-2004 * Low GPA | 1.077* | 1.108* | 1.079 | 0.971 | 1.056 |
|  | [1.000,1.159] | [1.025,1.199] | [0.875,1.331] | [0.880,1.070] | [0.943,1.182] |
| 2005-2010* Low GPA | 1.164*** | 1.255*** | 0.807 | 1.135** | 0.953 |
|  | [1.085,1.250] | [1.165,1.353] | [0.651,1.000] | [1.033,1.247] | [0.856,1.062] |
| N (students) | 2 252 703 | 2 077 489 | 175 158 | 1 099 819 | 1 152 850 |
| N (person-years) | 17 799 856 | 16 444 084 | 1 355 382 | 8 671 046 | 9 128 563 |

Table reports hazard ratios, with 95% confidence intervals in brackets. * p<0.05, ** p<0.01, *** p<0.001. Abbreviations: ref = reference category; GPA = Grade point average

**Table G3 – Discrete time proportional hazard models with internalizing disorders as the outcome. 30 % as cutoff for low GPA**

|  | Column 1  Full sample | Column 2  Native-born | Column 3  Immigrant | Column 4  Girls | Column 5  Boys |
| --- | --- | --- | --- | --- | --- |
| *Graduation year (ref: 1990-1997)* |  |  |  |  |  |
| 1998-2004 | 1.837*** | 1.883*** | 1.303*** | 1.877*** | 1.759*** |
|  | [1.757,1.921] | [1.797,1.973] | [1.126,1.508] | [1.780,1.980] | [1.620,1.910] |
| 2005-2010 | 2.335*** | 2.403*** | 1.636*** | 2.242*** | 2.590*** |
|  | [2.236,2.437] | [2.297,2.514] | [1.405,1.906] | [2.128,2.361] | [2.397,2.799] |
| *GPA (ref: high/medium GPA)* |  |  |  |  |  |
| Low GPA | 2.065*** | 2.090*** | 1.563*** | 2.444*** | 2.222*** |
|  | [1.965,2.169] | [1.983,2.202] | [1.348,1.814] | [2.297,2.601] | [2.044,2.415] |
| *Graduation year * GPA* |  |  |  |  |  |
| 1998-2004 * Low GPA | 1.018 | 1.024 | 1.118 | 0.990 | 1.061 |
|  | [0.955,1.084] | [0.958,1.095] | [0.922,1.356] | [0.914,1.071] | [0.954,1.180] |
| 2005-2010* Low GPA | 1.057 | 1.082* | 0.943 | 1.082* | 0.961 |
|  | [0.995,1.124] | [1.015,1.153] | [0.772,1.151] | [1.001,1.168] | [0.869,1.063] |
| N (students) | 2 252 703 | 2 077 489 | 175 158 | 1 099 819 | 1 152 850 |
| N (person-years) | 17 799 856 | 16 444 084 | 1 355 382 | 8 671 046 | 9 128 563 |

Table reports hazard ratios, with 95% confidence intervals in brackets. * p<0.05, ** p<0.01, *** p<0.001. Abbreviations: ref = reference category; GPA = Grade point average

**Additional file H. Alternative operationalizations of graduation years**

Additional file H investigates alternative ways of measuring time, that is, graduation years or cohorts. Table H1 and H2 shows results with graduation years entered as a continuous variable in the models. Graduation years have been recoded and centered at 1990, meaning that 0 represents 1990, 1 represents 1991 and so on. The association between low GPA and internalizing disorders became linearly stronger over time for the sample as whole as well as for native-born youth and girls, as indicated by the significant interaction terms.

Table H3 shows results with both graduation years and GPA percentiles entered as continuous variables. The linear interaction between graduation year and GPA is significant and negative for the sample as a whole as well as for native-born youth and girls.

Table H4 and H5 show results with an alternative categorization of graduation years, with 1990-1997 as reference category and later cohorts grouped into four instead of two approximately equally sized categories: 1998-2001, 2002-2004, 2005-2007 and 2008-2010. The results are similar to the main results, with larger and more often significant interaction terms for the later graduation cohorts.

**Table H1. Discrete time proportional hazard models with internalizing disorders as the outcome. Continuous measure of graduation year**

|  | Column 1  Full sample | Column 2  Native-born | Column 3  Immigrant | Column 4  Girls | Column 5  Boys |
| --- | --- | --- | --- | --- | --- |
| *Graduation year* |  |  |  |  |  |
| Graduation year | 1.059*** | 1.061*** | 1.034*** | 1.055*** | 1.071*** |
|  | [1.057,1.062] | [1.059,1.064] | [1.025,1.044] | [1.052,1.058] | [1.066,1.075] |
| *GPA (ref: high/medium GPA)* |  |  |  |  |  |
| Low GPA | 2.171*** | 2.192*** | 1.735*** | 2.486*** | 2.608*** |
|  | [2.057,2.291] | [2.070,2.322] | [1.472,2.044] | [2.319,2.665] | [2.389,2.847] |
| *Graduation year * GPA* |  |  |  |  |  |
| Graduation year * Low GPA | 1.007*** | 1.009*** | 0.995 | 1.008** | 0.995 |
|  | [1.003,1.011] | [1.005,1.013] | [0.983,1.008] | [1.003,1.013] | [0.988,1.001] |
| N (students) | 2 252 703 | 2 077 489 | 175 158 | 1 099 819 | 1 152 850 |
| N (person-years) | 17 799 856 | 16 444 084 | 1 355 382 | 8 671 046 | 9 128 563 |

Table reports hazard ratios, with 95% confidence intervals in brackets. * p<0.05, ** p<0.01, *** p<0.001. Abbreviations: ref = reference category; GPA = Grade point average

**Table H2. Discrete time proportional hazard models with internalizing disorders as the outcome. Continuous measure of graduation year**

|  | Column 1  Full sample | Column 2  Native-born | Column 3  Immigrant | Column 4  Girls | Column 5  Boys |
| --- | --- | --- | --- | --- | --- |
| *Graduation year* |  |  |  |  |  |
| Graduation year | 1.060*** | 1.063*** | 1.030*** | 1.055*** | 1.069*** |
|  | [1.058,1.063] | [1.060,1.066] | [1.021,1.040] | [1.052,1.059] | [1.065,1.074] |
| *UPS completion (ref: completed UPS)* |  |  |  |  |  |
| Not completed UPS | 3.929*** | 4.060*** | 2.656*** | 3.947*** | 4.289*** |
|  | [3.705,4.167] | [3.815,4.321] | [2.221,3.177] | [3.665,4.250] | [3.896,4.722] |
| *Graduation year * UPS completion* |  |  |  |  |  |
| Graduation year * Not completed UPS | 1.007*** | 1.009*** | 1.001 | 1.014*** | 1.000 |
|  | [1.003,1.012] | [1.004,1.014] | [0.987,1.015] | [1.008,1.019] | [0.993,1.007] |
| N (students) | 2 235 735 | 2 065 463 | 170 233 | 1 090 426 | 1 145 287 |
| N (person-years) | 13 100 112 | 12 117 627 | 982 312 | 6 372 269 | 6 727 750 |

Table reports hazard ratios, with 95% confidence intervals in brackets. * p<0.05, ** p<0.01, *** p<0.001. Abbreviations: ref = reference category; UPS = Upper secondary school.

**Table H3. Discrete time proportional hazard models with internalizing disorders as the outcome. Continuous measure of graduation year and of GPA.**

|  | Column 1  Full sample | Column 2  Native-born | Column 3  Immigrant | Column 4  Girls | Column 5  Boys |
| --- | --- | --- | --- | --- | --- |
| *Graduation year* |  |  |  |  |  |
| Graduation year | 1.0679*** | 1.0732*** | 1.0262*** | 1.0660*** | 1.0650*** |
|  | [1.0642,1.0715] | [1.0693,1.0771] | [1.0161,1.0363] | [1.0614,1.0707] | [1.0592,1.0708] |
| *GPA* |  |  |  |  |  |
| GPA percentile | 0.9866*** | 0.9868*** | 0.9876*** | 0.9845*** | 0.9811*** |
|  | [0.9855,0.9876] | [0.9857,0.9879] | [0.9843,0.9909] | [0.9833,0.9857] | [0.9791,0.9831] |
| *Graduation year * GPA* |  |  |  |  |  |
| Graduation year * GPA percentile | 0.9999*** | 0.9998*** | 1.0002 | 0.9999** | 1.0001 |
|  | [0.9998,0.9999] | [0.9997,0.9999] | [1.0000,1.0005] | [0.9998,0.9999] | [0.9999,1.0002] |
| N (students) | 2 252 703 | 2 077 489 | 175 158 | 1 099 819 | 1 152 850 |
| N (person-years) | 17 799 856 | 16 444 084 | 1 355 382 | 8 671 046 | 9 128 563 |

Table reports hazard ratios, with 95% confidence intervals in brackets. * p<0.05, ** p<0.01, *** p<0.001. Abbreviations: ref = reference category; GPA = Grade point average

**Table H4. Discrete time proportional hazard models with internalizing disorders as the outcome. More fine-grained categorization of graduation years.**

|  | Column 1  Full sample | Column 2  Native-born | Column 3  Immigrant | Column 4  Girls | Column 5  Boys |
| --- | --- | --- | --- | --- | --- |
| *Graduation year (ref: 1990-1997)* |  |  |  |  |  |
| 1998-2001 | 1.641*** | 1.665*** | 1.297*** | 1.686*** | 1.558*** |
|  | [1.564,1.721] | [1.584,1.752] | [1.119,1.503] | [1.592,1.785] | [1.430,1.698] |
| 2002-2004 | 1.982*** | 2.052*** | 1.295** | 1.994*** | 2.003*** |
|  | [1.890,2.079] | [1.952,2.157] | [1.104,1.520] | [1.883,2.112] | [1.839,2.181] |
| 2005-2007 | 2.247*** | 2.324*** | 1.507*** | 2.181*** | 2.468*** |
|  | [2.149,2.350] | [2.218,2.435] | [1.281,1.773] | [2.064,2.304] | [2.284,2.667] |
| 2008-2010 | 2.369*** | 2.442*** | 1.688*** | 2.215*** | 2.785*** |
|  | [2.264,2.478] | [2.330,2.559] | [1.436,1.985] | [2.096,2.342] | [2.577,3.009] |
| *GPA (ref: high/medium GPA)* |  |  |  |  |  |
| Low GPA | 2.214*** | 2.258*** | 1.602*** | 2.626*** | 2.435*** |
|  | [2.105,2.330] | [2.139,2.384] | [1.381,1.857] | [2.459,2.805] | [2.244,2.644] |
| *Graduation year * GPA* |  |  |  |  |  |
| 1998-2001 * Low GPA | 1.007 | 1.020 | 1.034 | 0.950 | 1.052 |
|  | [0.933,1.086] | [0.941,1.107] | [0.830,1.287] | [0.861,1.047] | [0.929,1.190] |
| 2002-2004 * Low GPA | 1.083* | 1.090* | 1.222 | 1.013 | 1.067 |
|  | [1.004,1.168] | [1.006,1.181] | [0.967,1.544] | [0.919,1.117] | [0.944,1.206] |
| 2005-2007 * Low GPA | 1.145*** | 1.173*** | 1.050 | 1.119* | 1.033 |
|  | [1.066,1.229] | [1.088,1.264] | [0.830,1.327] | [1.020,1.228] | [0.923,1.157] |
| 2008-2010 * Low GPA | 1.092* | 1.133** | 0.873 | 1.130* | 0.889* |
|  | [1.015,1.174] | [1.050,1.222] | [0.688,1.108] | [1.029,1.241] | [0.793,0.998] |
| N (students) | 2 252 703 | 2 077 489 | 175 158 | 1 099 819 | 1 152 850 |
| N (person-years) | 17 799 856 | 16 444 084 | 1 355 382 | 8 671 046 | 9 128 563 |

Table reports hazard ratios, with 95% confidence intervals in brackets. * p<0.05, ** p<0.01, *** p<0.001. Abbreviations: ref = reference category; GPA = Grade point average.

**Table H5. Discrete time proportional hazard models with internalizing disorders as the outcome. More fine-grained categorization of graduation years.**

|  | Column 1  Full sample | Column 2  Native-born | Column 3  Immigrant | Column 4  Girls | Column 5  Boys |
| --- | --- | --- | --- | --- | --- |
| *Graduation year (ref: 1990-1997)* |  |  |  |  |  |
| 1998-2001 | 1.701*** | 1.730*** | 1.321*** | 1.714*** | 1.674*** |
|  | [1.619,1.787] | [1.642,1.823] | [1.135,1.538] | [1.612,1.823] | [1.539,1.821] |
| 2002-2004 | 2.063*** | 2.138*** | 1.344*** | 2.028*** | 2.125*** |
|  | [1.964,2.167] | [2.031,2.252] | [1.141,1.582] | [1.908,2.156] | [1.956,2.308] |
| 2005-2007 | 2.299*** | 2.395*** | 1.411*** | 2.157*** | 2.576*** |
|  | [2.195,2.409] | [2.282,2.515] | [1.203,1.655] | [2.034,2.287] | [2.387,2.780] |
| 2008-2010 | 2.392*** | 2.474*** | 1.643*** | 2.222*** | 2.724*** |
|  | [2.282,2.508] | [2.355,2.599] | [1.387,1.947] | [2.093,2.359] | [2.521,2.943] |
| *UPS completion (ref: completed UPS)* |  |  |  |  |  |
| Not completed UPS | 4.227*** | 4.411*** | 2.566*** | 4.314*** | 4.502*** |
|  | [4.007,4.459] | [4.168,4.669] | [2.186,3.012] | [4.033,4.616] | [4.126,4.913] |
| *Graduation year * UPS completion* |  |  |  |  |  |
| 1998-2001 * Not completed UPS | 0.914* | 0.926 | 0.976 | 0.949 | 0.887 |
|  | [0.843,0.992] | [0.849,1.009] | [0.766,1.244] | [0.856,1.052] | [0.777,1.013] |
| 2002-2004 * Not completed UPS | 1.020 | 1.017 | 1.246 | 1.054 | 0.988 |
|  | [0.941,1.106] | [0.934,1.108] | [0.970,1.602] | [0.951,1.169] | [0.867,1.125] |
| 2005-2007 * Not completed UPS | 1.067 | 1.061 | 1.269 | 1.204*** | 0.908 |
|  | [0.989,1.151] | [0.980,1.149] | [0.988,1.629] | [1.093,1.327] | [0.804,1.026] |
| 2008-2010 * Not completed UPS | 1.096* | 1.140** | 0.808 | 1.197*** | 0.973 |
|  | [1.013,1.184] | [1.051,1.237] | [0.616,1.059] | [1.082,1.323] | [0.860,1.101] |
| N (students) | 2 235 735 | 2 065 463 | 170 233 | 1 090 426 | 1 145 287 |
| N (person-years) | 13 100 112 | 12 117 627 | 982 312 | 6 372 269 | 6 727 750 |

Table reports hazard ratios, with 95% confidence intervals in brackets. * p<0.05, ** p<0.01, *** p<0.001. Abbreviations: ref = reference category; UPS = Upper secondary school.

**References for the online supplementary materials**

Allison, P. D. (1982). Discrete-time methods for the analysis of event histories. Sociological Methodology, 13, 61-98.

Breen, R., Karlson, K. B., & Holm, A. (2018). Interpreting and Understanding Logits, Probits, and Other Nonlinear Probability Models. *Annual Review of Sociology*, *44*(1), 39-54.

Hernán, M. A. (2010). The Hazards of Hazard Ratios. Epidemiology, 21(1), 13-15.

Mood, C. (2010). Logistic Regression: Why We Cannot Do What We Think We Can Do, and What We Can Do About It. *European Sociological Review*, *26*(1), 67-82. [06](https://doi.org/10.1093/esr/jcp006)

Stensrud, M. J., & Hernán, M. A. (2020). Why Test for Proportional Hazards? JAMA, 323(14), 1401-1402.
